# Rapid Detection of Deoxynivalenol in Dry Pasta Using a Label-Free Immunosensor

**DOI:** 10.3390/bios12040240

**Published:** 2022-04-13

**Authors:** Francesca Malvano, Roberto Pilloton, Alfredo Rubino, Donatella Albanese

**Affiliations:** 1 Department of Industrial Engineering, University of Salerno, 84084 Fisciano, SA, Italy; fmalvano@unisa.it (F.M.); arubino@unisa.it (A.R.); 2 Institute of Crystallography of the National Research Council (CNR), Department of Chemistry and Material Technology, 00015 Montelibretti, RA, Italy; roberto.pilloton@cnr.it

**Keywords:** biosensors, antibody, mycotoxin, differential pulse voltammetry, gold electrode, PAMAM

## Abstract

This work focused on the development and optimization of an impedimetric label-free immunosensor for detecting deoxynivalenol (DON). A monoclonal antibody for DON detection was immobilized on a modified gold electrode with a cysteamine layer and polyamidoamine (PAMAM) dendrimers. Cyclic voltammetry and electrochemical impedance spectroscopy techniques were used to monitor the layer-by-layer development of the immunosensor design, while electrochemical impedance spectroscopy and differential pulse voltammetry were employed to investigate the antigen/antibody interaction. The PAMAM dendrimers, allowing to immobilize a large number of monoclonal antibodies, permitted reaching, through the DPV technique, a high sensitivity and a low limit of detection equal to 1 ppb. The evaluation of the possible reuse of the immunosensors highlighted a decrease in the analytical performances of the regenerated immunosensors. After evaluating the matrix effect, the developed immunosensor was used to quantify DON in pasta samples spiked with a known mycotoxin concentration. Taking into consideration the DON extraction procedure used for the pasta samples and the matrix effect related to the sample, the proposed immunosensor showed a limit of detection of 50 ppb, which is lower than the maximum residual limit imposed by European Regulation for DON in dry pasta (750 ppb).

## 1. Introduction

Mycotoxins are toxic metabolites produced by fungi both in the field and during the production, transport, transformation, and storage of food products. They are a significant concern for human and animal diseases and for the economic effects due to crop contamination [1].

Mycotoxin contamination affects approximately 25% of all food crops each year, causing significant financial loss worldwide. Therefore, due to their extensive economic and health risks, the presence of mycotoxins in feed and food products has been widely investigated for years [2]. One of the most significant mycotoxins in the world is deoxynivalenol, commonly known as vomitoxin.

Deoxynivalenol (DON) is a mycotoxin produced by *Fusarium graminearum* and *Fusarium culmorum* pathogens commonly present in cereal grains. It commonly contaminates agricultural raw materials, and it is commonly found in food crops such as wheat, maize, rye, barley, and other cereals worldwide, making it a potential hazard for human health [3].

Chemically it belongs to the group of trichothecenes, sesquiterpene mycotoxins characterized by a 12,13-epoxy group.

DON is a very stable compound, resistant to thermal treatments of feed and food products [4]. Like other trichothecenes, DON is responsible for inhibiting protein synthesis. Ingestion by animals and humans of highly contaminated products can lead to serious gastrointestinal diseases such as vomiting and bloody diarrhea. A long-term dietary exposure to DON involves, among the most serious effects, anorexia and impaired nutritional efficiency. Recently, it was also shown that low concentrations of DON could inhibit programmed cell death such as apoptosis induced by abiotic stress in *Arabidopsis* cell cultures [5].

To protect human and animal health, European countries have established, in Regulation (EC) No. 1881/2006, the maximum amount of DON in both feed and food products: the maximum residue limits set in different food products range from 200 to 1750 μg/kg depending on the nature of the cereals and cereal-based products. In the United States, the Food and Drug Administration has set advisory levels for DON in finished wheat products for human consumption (1000 μg/kg) and for grains and grain byproducts used for animal feed (5000 or 10,000 μg/kg depending on the species). Therefore, highly selective analytical techniques must be applied to ensure that DON concentrations in food and feed observe the legal regulations.

Today, the detection of deoxynivalenol in food products mainly occurs through chromatographic methods, particularly gas chromatography coupled with mass spectrometry [6], thin-layer chromatography [7], and high-performance liquid chromatography [8], with a limit of detection ranging from 0.10–40 ppb for pasta samples.

The merit of chromatographic methods is that they allow sensitive measurements of mycotoxins. However, on the other hand, they are also time-consuming and require complex treatments of samples before the analysis, high-level instrumentation, and extremely skilled personnel [9]. Moreover, enzyme-linked immunosorbent assays, based on an antibody’s capability to specifically bind DON, are commercially available and represent the most commonly used methods thanks to the low equipment required, simplicity, and high sensitivity, characterized by a limit of detection ranging from 30 to 80 ppb. However, they present shortcomings such as a time-consuming nature and cross-reactivity versus DON metabolites and other type of trichothecenes, NX toxins. A recent study [10] pointed out the development of an anti-DON monoclonal antibody with higher selectivity compared to a commercially available ELISA kit, highlighting that the accuracy of immunoassay-based techniques is, thus, strongly dependent on the selectivity of the monoclonal antibody employed. Electrochemical immunosensors are attractive for toxin and mycotoxin detection thanks to their numerous advantages such as operational simplicity, high sensitivity, low cost, and portability on-field [11]. 

Labeled immunosensors for DON detection have been developed in recent years. Using the DPV transduction technique, Qing et al. (2016) [9] reached a low limit of detection (LOD) equal to 0.005 ng/mL, while Zhilei et al. (2011) [12], through the electrochemical impedance spectroscopy transduction technique, developed a device capable of detecting DON in the range 0.001–0.3 ng/mL. An impedimetric immunosensor for direct DON quantification was proposed and tested on corn, wheat, and roasted coffee by Sunday et al. (2015) [13] with excellent results; the change in total impedance was proportional to DON amounts in the range of 6–30 ng/mL with a detection limit of 300 ng/mL. Lu et al. (2016) [14] reported an electrochemical immunosensor for rapid and sensitive detection of two mycotoxins simultaneously, fumonisin B1 and DON, on a screen-printed carbon electrode appropriately modified. The sensor showed high sensitivity, a low LOD equal to 8.6 ng/mL, and an irrelevant matrix effect when tested using extracts obtained from spiked corn samples.

In more recent years, different electrochemical approaches for signal amplification have been tested to reach lower limits of detection established by regulation; hydrogel materials [15], single-walled carbon nanotubes [9], recombinant Fab-fragment [16], gold nanoparticle-dotted 4-nitrophenylazo-functionalized graphene [13], and a fullerene/ferrocene/ionic liquid composite [12] were used in the immunosensor field to improve sensitivity. Among them, polyamidoamine dendrimers (PAMAM) were used mainly for the biofunctionalization of electrodes to increase the sensitivity and reach a low detection limit [17], before developing analytical devices able to detect almost the maximum residual limit for toxins and mycotoxins established by regulation.

In this study, an electrochemical immunosensor for label-free detection of DON in pasta samples was developed on a gold electrode functionalized with a cysteamine layer and PAMAM molecules.

Cyclic voltammetry (CV) and electrochemical impedance spectroscopy (EIS) techniques were applied to monitor the changes during stepwise modification of the electrode, while differential pulse voltammetry (DPV) was used to study the analytical performance of the developed immunosensor. After evaluating any matrix effects, the immunosensor, under optimized operative conditions, was used to detect DON amounts in different samples of pasta, the most common food product that can be affected by DON contamination. Lastly, the possible reuse of the DON immunosensor was also investigated.

## 2. Materials and Methods

### 2.1. Reagents

Cysteamine, glutaraldehyde, and polyamidoamine (PAMAM) dendrimer generation 4 (ethylenediamine core) were obtained from Merk (Germania), as well as ethanolamine, methanol, and sulfuric acid.

Potassium hexacyanoferrate (III) and potassium ferrocyanide were obtained from Carlo Erba Reagents (Milano, Italy). The monoclonal antibody for deoxynivalenol detection was obtained from LaboSpace Srl (Milano, Italy), while the deoxynivalenol solution (5 g/mL) was purchased from Merck.

### 2.2. Experimental Measurements and Apparatus

Each step of electrode modification for the immunosensor development was monitored by CV and EIS transduction techniques.

The voltametric measurements were performed at room temperature in a solution of 1 mM ferri/ferrocyanide redox couple (K_3_[Fe(CN)_6_]/K_4_[Fe(CN)_6_], 1:1) in PB 0.1 M pH 7.4, by cycling from −0.6 to 0.6 V vs. the reference electrode with a scan rate of 0.05 mV/s.

For the impedimetric measurements, a variation range of 10 mV was imposed to 0.00 mV (vs. the reference electrode) DC potential in the range of frequency from 0.1 to 10^4^ Hz. The impedimetric data were plotted in Nyquist plots, where the impedance is displayed as the sum of the real component (Z^I^) and the imaginary component (Z^II^). All measurements were performed in the redox solution previously described. All the impedimetric results were fitted with the common Randles circuit, as reported in our previous studies [18,19,20]. 

DPV was employed to investigate the antigen/antibody interaction with the following operative conditions: potential range −1.0 to 0.5 V, scan speed 20 mV/s, pulse repetition 0.1 s, and pulse amplitude 20 mV. The redox solution used for DPV was the same for CV and EIS measurements. 

Electrochemical measurements were performed with an AUTOLAB PGSTAT 204 potentiostat (Metrohm Technology, Switzerland) with the impedance module (FRA32M). Nova software (Methrom) was used to analyze the experimental results. 

A thin-film gold microelectrode array (Micrux Technologies, Spain) was used for the immunosensor construction, with a 1 mm diameter working electrode.

For DON analysis, 100 μL of DON at different amounts was dropped onto the working electrode of the immunosensor and incubated for 30 min to allow the formation of antibody/antigen complexes. Before the EIS and DPV measurements, the immunosensor was rinsed thoroughly with abundant amounts of PB. All measurements were performed at room temperature.

### 2.3. Design of the Immunosensor for the Detection of DON 

Before the modification, gold electrodes were subjected to 10 cycles in 0.05 M sulfuric acid solution in a range from −1.0 to +1.3 V at 100 mV/s scan rate. 

The surface of the working electrode was modified by electrodeposition of a layer of cysteamine, following the procedure reported by Malvano et al. (2017) [18]. 

PAMAM dendrimers were anchored on a cysteamine-modified electrode surface after the activation of cysteamine with glutaraldehyde solution 5% (*v*/*v*). Then, glutaraldehyde solution 5% (*v*/*v*) was dropped onto the PAMAM-modified electrode for 1 h and rinsed with PB. Next, monoclonal anti-DON (1 mg/mL) was dropped onto the electrodes. Finally, the PAMAM unreacted active sites were blocked with 1 M ethanolamine solution. The electrode was rinsed with PB at the end of each modification step.

**Figure 1:**
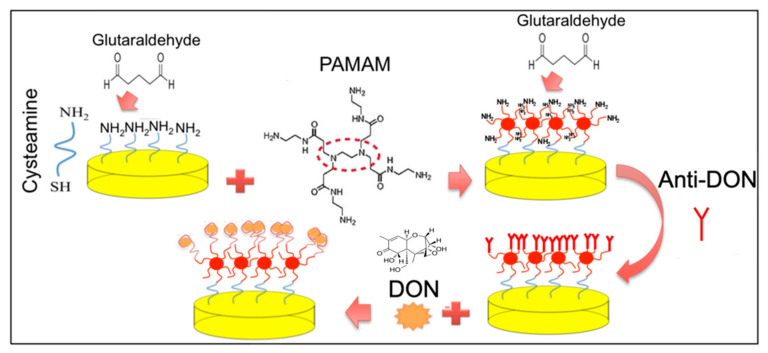
Immunosensor fabrication steps followed by the measurement step.

The immunosensor fabrication steps followed by the measurement step are illustrated in Figure 1.

### 2.4. Extraction of DON from Italian Dry Pasta

The extraction of DON from pasta was performed according to Raiola et al. (2012) [21] with some modifications. Briefly, 2 g of finely ground pasta spiked with different amounts of DON (50 ppb, 250 ppb, 750 ppb, 1500 ppb, 3000 ppb, 6000 ppb) was mixed with 100 mL of a methanol/water (70:30) solution and extracted by agitation on a magnetic stirrer for 1 h. The samples were then centrifuged at 3000 rpm at 25 °C for 10 min; then, 100 μL of the resulting extract was analyzed.

## 3. Results and Discussion

### 3.1. Characterization of the Electrode Surface

The surface characterization of the Au electrode for the preparation of the DON immunosensor was carried out using CV and ElS, which provide helpful information on the electrical behavior of the electrode surface during its functionalization with cysteamine and PAMAM dendrimers.

**Figure 2:**
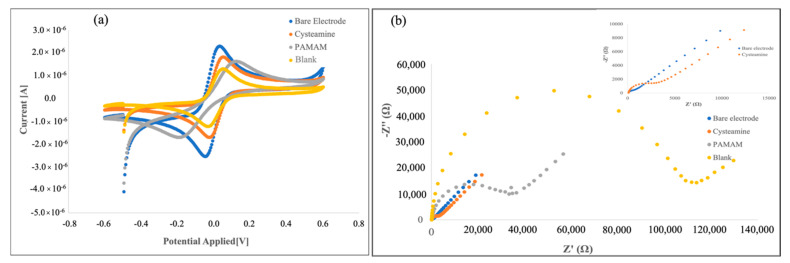
Cyclic voltammograms (**a**) and Nyquist plots (**b**) after each step of DON immunosensor construction. Inset: Nyquist plots of bare and cysteamine electrode.

According to our previous work [18], the formation of the cysteamine layer, followed by PAMAM immobilization and monoclonal antibody binding, causes a decrease in the anodic and cathodic peaks of the CVs, presumably due to the hindrance effect, of the different immobilization layers, on the electron transfer rate (Figure 2a).

Nyquist plots associated with each step of surface modification recorded an increase in the impedance of the system after each step of immobilization (Figure 2b), due to the layer coating on the working electrode surface that became thicker in a step-by-step manner [19]. 

Furthermore, the increase in impedance observed in the last step points out that the immobilization of the antibody on the electrode surface was successful. PAMAM G4 dendrimers, with their many advantages associated with the structural homogeneity and the high density of functional chain end groups, allow the anchoring of a high number of antibody molecules on the sensing surface [22], as evidenced by the significant increase in the resulted impedimetric response. 

### 3.2. Analytical Performances of Immunosensor

With the aim of identifying a better electrochemical method able to reach a higher analytical performance of the biosensor, the fabricated electrochemical immunosensor was evaluated by EIS and DPV. Different responses of EIS and DPV under different amounts of DON were obtained. 

**Figure 3:**
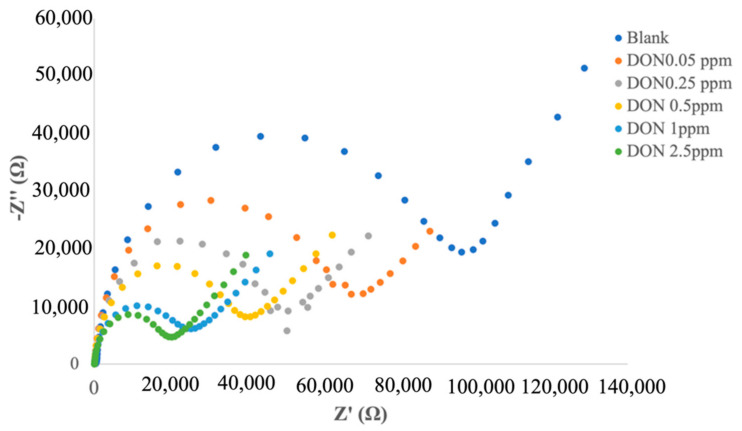
EIS responses of immunosensor to different amounts of DON.

When the immunosensor bound with increasing amount of DON, a decrease in the semicircular diameters of Nyquist plots was registered (Figure 3). In particular, by fitting the impedimetric results with the common Randles circuit, a decrease in the charge transfer resistance R_ct_ was obtained with the increase in DON concentration.

The calibration curve was obtained by plotting the logarithmic value of DON concentration versus Δ*R_ct_*, calculated as follows:

(1) $$\Delta R_{ct}\%=\frac{R_{ct}{}_{\;Blank-}R_{ctDON}}{R_{ct\;Blank}}\cdot 100,$$

where $R_{ct}{}_{\;Blank\;}$ is the *R_ct_* value after the immobilization of anti-DON, and $R_{ctDON}$ is the *R_ct_* after the immunocomplex.

**Figure 4:**
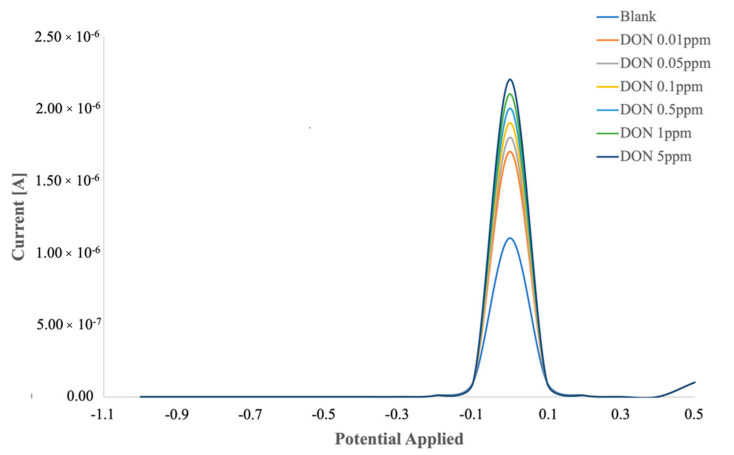
DPV responses of immunosensor to different amounts of DON.

When the DPV technique was used to evaluate the response due to the immunocomplex, an increase in peak height with the increase in DON concentration was registered (Figure 4).

The calibration curve was obtained by plotting the logarithmic value of DON concentration versus the percentage variation in peak height, calculated as follows:

(2) $$\Delta H\%=\frac{H_{DON-H_{Blank}}}{H_{Blank}}\cdot 100,$$

where *H_Blank_* is the peak height value when anti-DON was immobilized on the electrode surface, and *H_DON_* is the peak height value after the binding between anti-DON and DON. 

**Figure 5:**
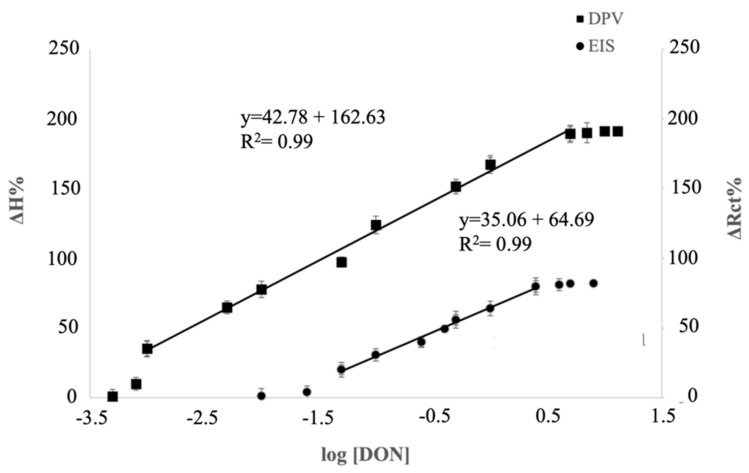
Calibration curve of DON immunosensor investigated through EIS (•) and DPV( ▪) transduction techniques.

The calibration curves of the immunosensors characterized with EIS and DPV versus different concentrations of DON (Figure 5) showed a lower detectable DON amount, as well as a wider linear range, when DPV was employed as transduction technique. A recent study [23], in which an electrochemical immunosensor for histidine-rich protein 2 was developed, the analytical measurement was carried out with both EIS and DPV, and no differences were observed in the linear range. Moreover, in that case, the electrochemical signal increased with the concentration in EIS, while it decreased in DPV.

These differences could be due to different immobilization techniques, the nature of the MAb, and the electrochemical operative conditions for DPV and EIS applied during the measurements.

Table 1 shows a comparison among the label-free immunosensors for DON detection reported in literature.

The data reported in Table 1 point out that the limit of detection, as well as the linear range of the immunosensors, is influenced by the immobilization and transduction technique applied for the study. Moreover, as reported in our previous studies [20,24], the linear range, sensitivity, and LOD parameters also depend on the amount of receptor immobilized on the electrode surface.

The use of PAMAM dendrimer allowed obtaining a very high immobilization yield, reaching the widest linear range among the DON immunosensors developed in the literature, allowing the developed biosensor to analyze a wide range of food products.

Moreover, considering the DON extraction procedure commonly used for pasta samples, the proposed immunosensor showed an LOD equal to 50 ppb, an amount much lower than the maximum residual limits imposed by the regulation for DON in dry pasta (750 ppb).

The reproducibility of the immunosensor performance was evaluated by calculating the relative standard deviation (%RSD) value on five different immunosensors characterized at 0.05 μg/mL. The RSD value equal to 3.80% confirmed its good reproducibility. 

The analysis time of the immunosensor is affected by the incubation time necessary to create the immunocomplex. In this study, the immunocomplex formation between DON and anti-DON required 30 min, while both electrochemical transductions, EIS and DPV, required only 1 min. Thus, the total time of the analysis was 31 min.

**Figure 6:**
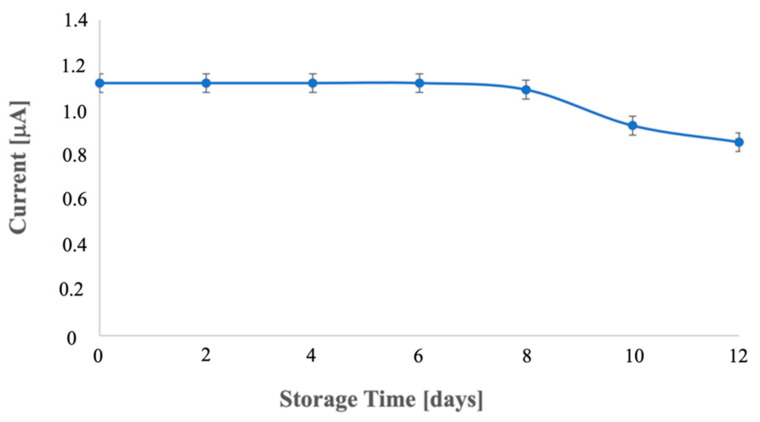
Storage stability of the immunosensor stored at 4 °C over 12 days. Error bars are standard deviations of three measurements.

Lastly, the immunosensor stability was determined by storing the immunosensor at 4 °C for 12 days without chemical preservatives and characterizing it at regular intervals. Over the first 6 days, the current signal remained stable; however, a gradual decline in response was observed from day 6 onward, until reaching a decrease of 23.22% on the 12th day (Figure 6). 

However, at 8 days of storage, the immunosensor showed negligible activity loss (2.31%).

Although the goal of this work was the development of a disposable device, the possible regeneration of the proposed immunosensors was investigated. For this aim, after the formation of the immunocomplex between antibodies and 5 ppb DON, the electrode was dipped into a solution of methanol, acetonitrile, and water (10:10:80) for 30 min. After this time, the immunosensor was washed and characterized with the previous DON amounts (5 ppb). 

**Figure 7:**
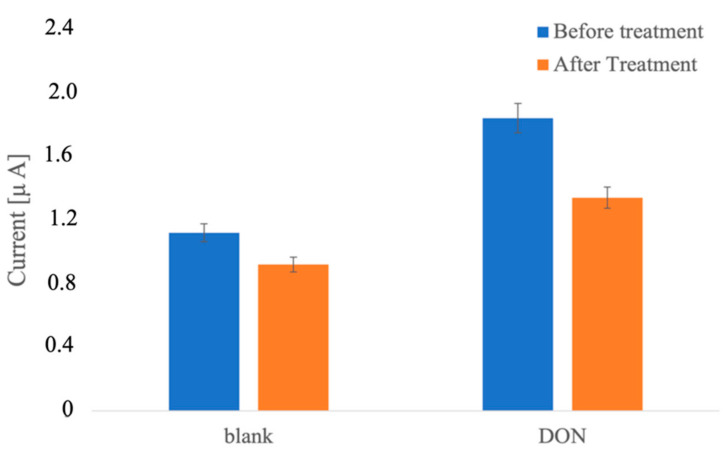
DPV responses (peak heights) before and after treatment in the detachment solution (methanol, acetonitrile, and water 10:10:80). Number of electrodes tested = 5.

The DPV response obtained after the pretreatment for antigen detachment (Figure 7) presented a lower peak height, highlighting a decrease in antibodies present on the electrode surface and, thus, a change in the analytical performances of the regenerated immunosensor.

The obtained results indicate that the developed DON immunosensor is a “single-use” device.

### 3.3. Immunosensor Performance in Extracts from Dry Pasta 

Six brands of Italian pasta were analyzed to study the possibility of applying the immunosensor to pasta samples, and the matrix effect was evaluated. As shown in Table 2, in all cases, the increment in DPV responses after the interaction of the immunosensor with the extracted solution was around 11%. It is appropriate to think that this variation was due to the matrix effect.

With the aim of verifying the reliability of the developed immunosensor as an analytical device for DON detection and analysis in food matrices, different amounts of DON were spiked in pasta samples. According to the results in Table 3, good recovery percentages were obtained in the range 98.75–102.60%. As reported by Nguyen et al. [10] the cross-reactivity of DON antibodies with DON acetylated forms is highly probable; thus, the quantification of the mycotoxin deoxynivalenol could be overestimated when DON metabolites (3-ADON, 15-ADON, and/or DON-3G) are present in the food. However, previous studies [25] also showed the potential cytotoxic effects of DON metabolites, suggesting that a toxic equivalent factor of >1 should be used for 15-ADON co-occurring with DON in food materials, whereas the equivalent factor for 3-ADON should be slightly lower than 1. Lastly, even if no cross-reactivity tests versus DON metabolites were carried out in this study, the development of the immunosensor based on anti-DON antibodies highlights its potential use as a screening method for the recognition of mycotoxin deoxynivalenol in foodstuff.

## 4. Conclusions

A label-free electrochemical immunosensor based on a cysteamine/PAMAM layer for antibody immobilization was developed to detect DON in dry pasta. PAMAM dendrimers allow the immobilization of a high number of antibodies, reaching a satisfactory sensitivity and a very low LOD. In fact, taking into consideration the DON extraction procedures used and the matrix effect related to the pasta samples, the proposed immunosensor showed an LOD equal to 50 ppb, an amount much lower than the maximum residual limits imposed by European legislation for DON in dry pasta (750 ppb).

## Figures and Tables

**Table 1:** Comparison of the electrochemical DON immunosensor developed in this work with others reported in the literature.

Sensor Assay		LOD (ppb)	Linear Range (ppb)	RSD (%)	Reference
Au/Gel ^1^/MAb	EIS	0.001	0.001–0.5	1.05	[15]
GC ^2^/^3^ AuNps/G/PhNO_2_/MAb	EIS	30	6–30	6.50	[13]
^4^ SPE/^5^ AuNPs/PPy/ErGO/MAb	DPV	8.6	50–1000	5.71	[14]
Au/Cys/PAMAM/MAb	EIS	50	50–2500	2.12	This work
Au/Cys/PAMAM/MAb	DPV	1	1–5000	3.80	This work

^1^ Gel: guanosine-based small-molecular hydrogel. ^2^ GC: glassy carbon electrode. ^3^ AuNPs/G/PhNO_2_: gold nanoparticle-dotted 4-nitrophenylazo-functionalized graphene. ^4^ SPE: screen-printed electrode. ^5^ AuNPs/PPy/ErGO: AuNPs and polypyrrole electrochemically reduced graphene oxide nanocomposite film.

**Table 2:** Matrix effect.

Sample	DPV Response (ΔH%)
1	11.78 ± 1.12
2	11.97 ± 0.89
3	12.35 ± 0.91
4	10.83 ± 1.01
5	11.94 ± 1.03
6	12.52 ± 0.99

**Table 3:** Determination of DON in pasta sample through the proposed immunosensor.

Spiked Amount (ppb)	Found Amount (ppb)	Recovery (%)
50	50.61 ± 1.23	98.79
250	243.49 ± 7.67	102.60
750	753.38 ± 9.45	99.55
1500	1501.18 ± 8.67	99.92
3000	3037.45 ± 10.34	98.75

## Data Availability

Not applicable.
